# Multiparametric deep learning tissue signatures for a radiological biomarker of breast cancer: Preliminary results

**DOI:** 10.1002/mp.13849

**Published:** 2019-11-22

**Authors:** Vishwa S. Parekh, Katarzyna J. Macura, Susan C. Harvey, Ihab R. Kamel, Riham EI‐Khouli, David A. Bluemke, Michael A. Jacobs

**Affiliations:** ^1^ The Russell H. Morgan Department of Radiology and Radiological Sciences The Johns Hopkins University School of Medicine Baltimore MD 21205 USA; ^2^ Sidney Kimmel Comprehensive Cancer Center The Johns Hopkins University School of Medicine Baltimore MD 21205 USA; ^3^ Department of Computer Science The Johns Hopkins University Baltimore MD 21208 USA; ^4^ Hologic Inc 36 Apple Ridge Rd Danbury CT 06810 USA; ^5^ Department of Radiology and Radiological Sciences University of Kentucky Lexington KY 40536 USA; ^6^ Department of Radiology University of Wisconsin School of Medicine and Public Health Madison WI 53726 USA

**Keywords:** autoencoders, breast, cancer, CNN, deep learning, diffusion, machine learning, magnetic resonance imaging, multiparametric MRI, tissue biomarkers, tissue signature vector

## Abstract

**Purpose:**

Deep learning is emerging in radiology due to the increased computational capabilities available to reading rooms. These computational developments have the ability to mimic the radiologist and may allow for more accurate tissue characterization of normal and pathological lesion tissue to assist radiologists in defining different diseases. We introduce a novel tissue signature model based on tissue characteristics in breast tissue from multiparametric magnetic resonance imaging (mpMRI). The breast tissue signatures are used as inputs in a stacked sparse autoencoder (SSAE) multiparametric deep learning (MPDL) network for segmentation of breast mpMRI.

**Methods:**

We constructed the MPDL network from SSAE with 5 layers with 10 nodes at each layer. A total cohort of 195 breast cancer subjects were used for training and testing of the MPDL network. The cohort consisted of a training dataset of 145 subjects and an independent validation set of 50 subjects. After segmentation, we used a combined SAE‐support vector machine (SAE‐SVM) learning method for classification. Dice similarity (DS) metrics were calculated between the segmented MPDL and dynamic contrast enhancement (DCE) MRI‐defined lesions. Sensitivity, specificity, and area under the curve (AUC) metrics were used to classify benign from malignant lesions.

**Results:**

The MPDL segmentation resulted in a high DS of 0.87 ± 0.05 for malignant lesions and 0.84 ± 0.07 for benign lesions. The MPDL had excellent sensitivity and specificity of 86% and 86% with positive predictive and negative predictive values of 92% and 73%, respectively, and an AUC of 0.90.

**Conclusions:**

Using a new tissue signature model as inputs into the MPDL algorithm, we have successfully validated MPDL in a large cohort of subjects and achieved results similar to radiologists.

## 1 Introduction

A new paradigm is emerging in radiology with the increased computational capabilities and deep learning algorithms. Deep learning networks (DLN) allow for the learning of radiological relationships between different tissue types and provide new methods to segment and/or classify high‐dimensional data sets.^1^, ^2^, ^3^, ^4^, ^5^, ^6^, ^7^, ^8^, ^9^ These DLN algorithms allow for accurate and reliable prediction of tissue types from the input images with the aim to improve the radiologist’s clinical decision support in different diseases^10^, ^11^, ^12^, ^13^, ^14^, ^15^, ^16^, ^17^, ^18^, ^19^ and have been recently reviewed.^20^, ^21^

In the multiparametric MRI setting, using conventional T_1_‐ and T_2_‐weighted images and advanced MRI parameters of diffusion‐weighted imaging (DWI) and dynamic contrast‐enhanced imaging (DCE) provide qualitative and quantitative information of different tissue types. These sequences provide a distinct contrast of various tissues to construct unique signatures that provide specific information about the composition of the tissue.^22^, ^23^, ^24^, ^25^, ^26^, ^27^, ^28^, ^29^ Experienced radiologists use the variable contrast from MRI to identify and recognize tissue signatures of normal from abnormal features. Advanced deep learning algorithms by attempting to mimic the radiologist may allow for more accurate tissue characterization.

Prior work in using deep learning methods for segmentation of breast tissue from breast MRI has been focused on glandular tissue, breast boundaries, and lesions.^30^, ^31^, ^32^, ^33^, ^34^, ^35^, ^36^ These methods have been limited to using only one or two breast MRI sequences and with complete annotated datasets of the tissue of interest. These reports used a variety of current state of the art methods for segmenting the breast lesions including, modified convolutional neural network (CNN)‐based (U‐Net), long short‐term memory (LSTM), and deep reinforcement learning.^33^, ^36^, ^37^, ^38^, ^39^, ^40^ Using a deep reinforcement learning, breast lesion segmentations were obtained using bounding boxes trained to detect breast lesions.^37^ Hybrid deep learning approaches have increased the accuracy (>0.75) of breast lesion segmentations using combinations of CNN with other methods such as saliency mapping^38^ with Q learning^6^ or LSTM.^33^, ^39^ Amit et al.^38^ developed a hybrid mass‐detection algorithm that combines unsupervised candidate detection with deep learning‐based classification and saliency maps. They used a fully convolutional network (U‐Net for segmentation) and training the network using a bounding box with and with lesions for each slice in 117 patients (75 malignant and 42 benign). The accuracy metric was sensitivity of 0.80. Chen et al. developed an end‐to‐end spatio‐temporal network based on modified CNN (U‐Net) coupled with recurrent neural networks (RNN), that is, a ConvLSTM^41^ to segment breast DCE MRI.^39^ They applied the method to a fully annotated breast MRI dataset of 73 patients (28 malignant, 45 benign) with 3 convLSTM networks and a fusion operator for the DCE images. For quantitative comparison, they calculated the dice coefficient and positive predictive value, which was, 0.76 and 0.74, respectively. Similarly, Zheng et al.^33^ extended the convLSTM by adding a dense layer (DC‐LSTM) to the network to extract more features from the DCE images and a ResNet for classification. They utilized two MRI sequences, a diffusion weighted imaging and DCE sequence acquired from 72 patients (27 malignant and 45 benign). The resulting segmentation and classification using the DC‐LSTM increased the accuracy from 0.62 to 0.84, when only using convLSTM alone. However, all of these methods required a full annotated dataset from the breast MRI.

Tissue characterization using conventional machine or deep learning algorithms requires accurate labels for all tissue types present in the dataset. This labeling requirement requires radiologist or trained personnel to draw segmentation masks or labels on the various tissues present in each image. This task of creating complete labeled datasets is time consuming, expensive, and impractical. Therefore, advanced machine learning algorithms are needed to learn tissue signatures from a minimally labeled dataset.

Sparse autoencoders (SAE) are unsupervised neural networks that learn an intrinsic representation of the input features by attempting to reconstruct it.^42^, ^43^, ^44^, ^45^ The activation of each node in the hidden layer of the SAE is specialized to activate in response to a very specific subset of input data by introducing a sparsity constraint. The unsupervised nature of SAE allows training on the complete set of tissue signatures (labeled and unlabeled). By stacking several SAEs, you can construct a deep neural network termed as a Stacked SAE (SSAE).^46^ This permits the pretraining of a multiparametric deep learning (MPDL) model using multiparametric breast tissue signatures across images from multiple MRI sequences, without prior knowledge of the variable underlying tissue types. The MPDL model can be fine‐tuned by adding a supervised classification layer (e.g., Softmax, support vector machine) at the end of SSAE for tissue segmentation and classification of breast tissue and lesions.

This work developed and implemented novel tissue signatures for input into the MPDL model to segment different breast tissue types, that is, normal fatty and glandular tissue and breast lesions.^47^ Subsequently, we classified the identified breast lesions into benign or malignant using machine learning and demonstrated that the MPDL outcomes are similar to radiologists. Finally, we validated the MPDL tissue signature model with an independent MRI dataset obtained from different magnets and imaging planes of breast cancer patients.

## 2 Materials And Methods

### 2.1 Clinical subjects

All studies were performed under the institutional guidelines for clinical research under a protocol approved by the Johns Hopkins University School of Medicine Institutional Review Board (IRB) and all HIPAA agreements. The IRB waived the requirement for informed consent for this retrospective study. We obtained an independent deidentified breast dataset of 50 subjects from the University of California, San Francisco (UCSF) not requiring informed consent of the subjects. There were 195 women in this study, broken into two groups of subjects.

The first group comprised 145 women imaged at our institution and the second group included 50 women imaged at a different institution. The mean age of the first group of 145 female patients who met our inclusion criteria was 52 ± 11 yr (range = 22–80). Of the 145 patients, there were 97 with malignant lesions, 44 with benign lesions, and 4 normal (no‐lesion) patients. Table 1 summarizes the distribution of the benign and malignant lesions. The 50 cases comprising the independent validation dataset were obtained from a Phase 3 clinical trial for women receiving neoadjuvant chemotherapy for locally advanced breast cancer, commonly referred to as the I‐SPY trial.^48^, ^49^, ^50^ We used only the baseline study (i.e., pretreatment) before initiation of the therapeutic regimen to test the MPDL model.

**Table 1:** Summary of demographic and clinical data.

Malignant characteristics	IDC	DCIS + IDC	IDC + ILC	ILC	Sarcomatoid
	N = 36 (37%)	N = 31 (32%)	N = 19 (20%)	N = 10 (10%)	N = 1 (1%)
Age, yr^a^	51 ± 11	53 ± 9	57 ± 9	61 ± 11	68
Phenotype					
Luminal A^b^	17	11	10	9	0
Luminal B^b^	6	8	5	1	0
HER2+^b^	4	3	1	0	0
Triple negative^b^	9	9	3	0	1

Benign characteristics	Benign breast tissue	Stable imaging	Fibroadenoma	ADH	ADH with lobular features	Papilloma	LCIS
aAge, yr	49 ± 10	51 ± 12	42 ± 13	59 ± 9	53 ± 6	52 ± 1	51

DCIS, ductal carcinoma in situ; ILC, invasive lobular carcinoma; LCIS, lobular carcinoma in situ; IDC, invasive ductal carcinoma, ADH, atypical ductal hyperplasia, HER2+, human epidermal growth factor receptor 2.

a Data are presented as mean ± (SD).

b Data are presented as number of cases.

### 2.2 Multiparametric MRI imaging protocol

We performed MRI scans from our institution on a 3‐Tesla magnet (Philips North America Corporation), using a dedicated phased array breast coil with the lying patient prone with gentle compression applied to the breast to reduce motion. MRI sequences comprised of bilateral axial fat suppressed (FS) T_2_WI spin echo (TR/TE = 5700/102 ms) and fast spoiled gradient echo (FSPGR) T_1_WI (TR/TE = 200/4.4, Field of View (FOV) = 256 × 256, slice thickness (ST), 4, 1 mm gap); axial diffusion‐weighted images (DWI) (TR/TE = 5000/90 ms,b = 0–800, FOV = 192 × 192,ST = 6 mm). The Apparent Diffusion Coefficient (ADC) of water maps was constructed from the DWI; and finally, axial pre‐ and postdynamic contrast‐enhanced (DCE) images FSPGR T_1_WI (TR/TE = 20/4, FOV = 512 × 512, ST = 3 mm) were obtained after intravenous administration of a Gadolinium (Gd‐DTPA) contrast agent [0.2 mL/kg(0.1 mmol/kg)]. The contrast agent was injected at a rate of 2 mL/s using a power injector, with MRI imaging beginning after a 10‐s delay with the acquisition of 14 phases. A 20 cc saline flush followed the contrast bolus. The DCE protocol included 2 min of high temporal resolution (15 s per acquisition) imaging to capture the wash‐in phase of contrast enhancement. A high spatial resolution scan for 2 min then followed, with additional high temporal resolution images (15 s per acquisition) for an additional 2 min to characterize the wash‐out slope of the kinetic curve for pharmacokinetics (PK) of the DCE (PK‐DCE).^51^ The total scan time for the entire protocol was <45 min.

### 2.3 Pharmacokinetic (PK) contrast enhancement parameters

Pharmacokinetic‐dynamic contrast enhancement can provide metrics of the vascularity of breast tissue. The PK‐DCE quantitative metrics derived were volume transfer constant (*K* ^trans^ (min^−1^)) and the fractional volume of the extracellular extravascular space (EVF (V*_e_*)).^51^

The independent validation breast MRI scans were acquired on different 1.5‐T magnets using a dedicated breast phased array coils from a multicenter ACRIN I‐SPY clinical trial.^48^, ^49^, ^50^ The validation MRIs were scanned unilaterally in the sagittal plane consisting of a T_1_‐weighted FS DCE sequence with three postcontrast time points (TR ≤ 20 ms, TE = 4.5 ms, flip angle ≤ 45°, FOV: 160–180, matrix size > 256 × 192, ST ≤ 2.5 mm).

### 2.4 Multiparametric image registration

The mpMRI was registered using a hybrid registration algorithm that combines three‐dimensional (3D) wavelet transformation for 3D reslicing and rescaling of the MRI volumes with a nonlinear affine transformation to minimize the loss of information in image transformations.^52^, ^53^ The precontrast image of the DCE dataset was used as the reference image for all the other images. The hybrid registration scheme consists of several steps to reslice and match each modality using a combination of both affine and wavelet registration steps. First, orthogonal reslicing is performed to equalize FOV, matrix sizes, and the number of slices using wavelet transformation. Second, angular resampling of the target data was performed to match the reference data. Finally, similarity transformation (scaling and translation) between the reference and resliced target volume was performed. After registration, the mean‐square‐error (MSE) and Dice Similarity (DS) between the reference and registered target volumes were calculated.

### 2.5 Multiparametric MRI tissue signature generation

The Eigenimage filter (EI) segmentation algorithm was used to segment the breast lesions from the postcontrast DCE image.^54^ The EI is a linear filter that maximizes the projection of a desired tissue (lesion tissue) while it minimizes the projection of undesired tissues (glandular tissue) onto a composite image called an Eigenimage.^55^ We defined the tissue signatures of normal glandular and fatty tissue and lesions using the EI filter described below. The EI corrects for partial volume effects, which allows for improved demarcation of all underlying features.^56^

### 2.6 Multiparametric deep learning tissue signatures

**Figure 1:**
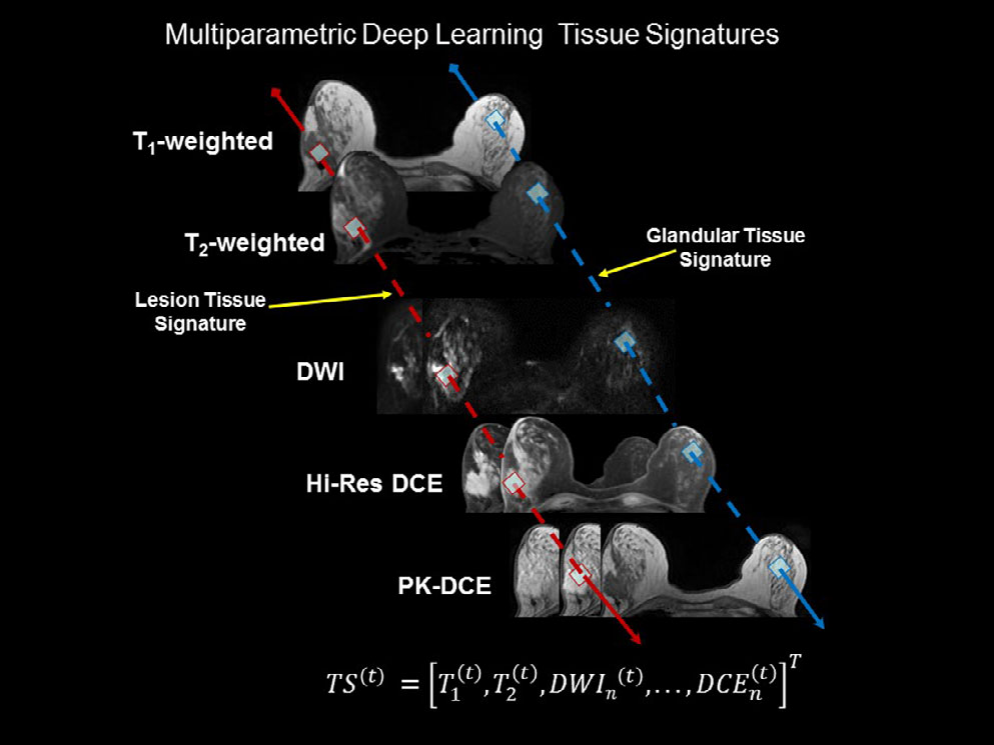
Demonstration of the tissue signatures (TS) defined on multiparametric breast MRI. Representative tissue signatures are shown for normal (glandular‐blue) and lesion tissue (red) from each input MRI. We define the tissue signature as a vector, TS^(ι)^, where (*ι*) (τ) defines the tissue type over each MRI image and n is the number of images each of respective sequence. There were T_1_‐weighted, T_2_‐weighted, high‐resolution (Hi‐Res) and pharmacokinetic (PK) dynamic contrast‐enhanced (DCE) images along with Diffusion Weighted Images (DWI) in the data set. [Color figure can be viewed at http://www.wileyonlinelibrary.com]

We trained the MPDL network on the breast tissue signatures defined by the EI filter from the MR images and these are shown in Fig. 1. The MPDL network builds a composite feature representation using the breast tissue signatures defined by the unique MRI tissue characteristics of the breast tissue. We define tissue signature vector as gray level intensity values corresponding to each voxel position within the images. Mathematically, the MPDL tissue signature vectors are defined as:

(1) $$MPDL\;Tissue\;Signature=TS^{\left(\tau \right)}={\left[T_{1}^{\left(t\right)},T_{2}^{\left(t\right)},DWI_{n}^{\left(t\right)},\cdots ,DCE_{n}^{\left(t\right)}\right]}^{T}$$

where, *(τ)* is the breast tissue type, *T* is the transpose, and *n,* the number of the images in the sequence. We defined tissue signatures for background, glandular, fatty, and lesion tissue. We create each set of MPDL tissue signature vectors automatically using a multiparametric region growing algorithm. We set the region growing tolerance at 5% standard deviation of the signal intensity of the surrounding pixels within the image. The final tissue signature’s region of interest (ROI) is then automatically created by computing a logical AND operation between the ROIs generated from selected region growing on each of the images. The user selects pixels based on the signal intensity within each image representing the desired tissue type, which forms a voxel that produces an ROI used for the tissue signature. For example, defining the glandular tissue signature voxel, the user selects representative pixels in a region of glandular tissue on the image. We show examples of glandular and lesion tissue signatures in Fig. 1. By using several images concurrently, the probability of a pixel from another tissue being included in the final ROI (due to noise, partial volume, and nonuniformities) is reduced.^56^ The computer time required for producing the final tissue signature ROI was less than a second for each tissue type within the breast.

### 2.7 Multiparametric segmentation deep learning network

#### 2.7.1 Stacked sparse autoencoder network architecture

**Figure 2:**
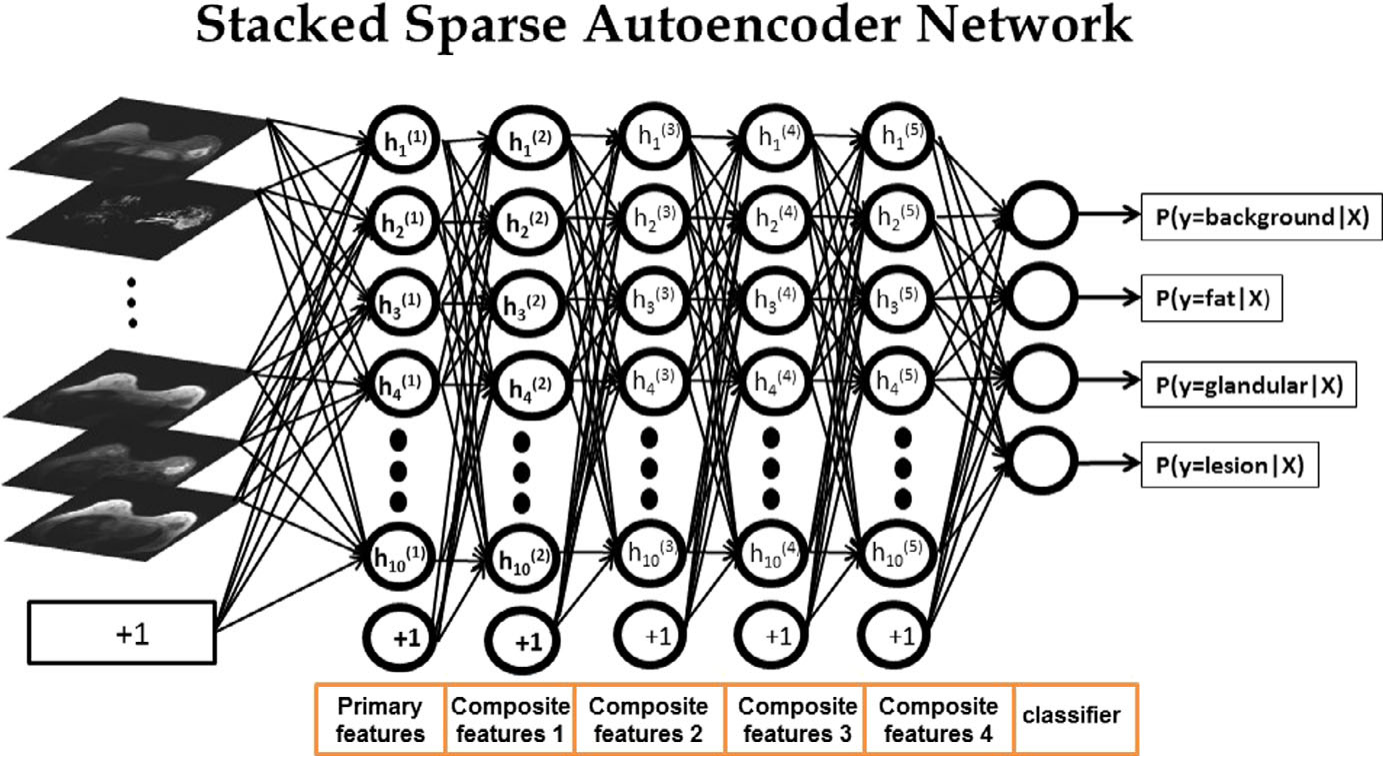
The framework for the multiparametric magnetic resonance imaging (mpMRI) segmentation deep learning network used to segment breast MRI. The stacked sparse encoder (SSAE) deep network architecture was constructed of five SAE hidden layers with ten nodes at each layer and a softmax classification layer that outputs the probability of different tissue types for the input tissue signature. The inputs into the SSAE are the tissue signatures defined from the mpMRI. The output is the segmented images for classification of the breast lesions. [Color figure can be viewed at http://www.wileyonlinelibrary.com]

We developed the mpMRI segmentation deep network using SSAE for segmenting a mpMRI breast dataset into different tissue types. Figure 2 shows the SSAE network architecture for mpMRI breast segmentation. Each SAE of the SSAE was pretrained by the tissue signature vectors to create a low‐dimensional representation of the input via the hidden layers. The input to each autoencoder except the first autoencoder was the hidden layer representation discovered by the previous layer and these encoding and decoding steps are fully detailed in the Appendix.^42^, ^43^, ^44^, ^45^ Briefly, the first layer of the autoencoder learns the low dimensional representation, $Y=\left\{y\left(1\right),y\left(2\right)\ldots ,y\left(N\right)\right\}\subset R^{d}$ from the training input tissue signatures, $X=\left\{x\left(1\right),x\left(2\right),\ldots ,x\left(N\right)\right\}\subset R^{D}$, where *D* is the dimensionality of the input tissue signatures, N is the total number of tissue signatures, and *d* is the number of nodes in the hidden layer of the autoencoder. The output of the first layer provides input for the subsequent layers. The output of the final sparse autoencoder was used for input to train a softmax classifier to identify tissue signatures as background, fat, glandular, or lesion.

#### 2.7.2 Multiparametric MRI segmentation deep network training and evaluation

For the MpMRI segmentation, the training parameters used from optimization were set as: the number of layers = 5, the number of nodes in each hidden layer = 10, L2 regularization penalty = 0.001, sparsity proportion = 0.25, sparsity regularization = 4.^44^, ^45^, ^57^ The transfer function for the autoencoder nodes was selected as the saturating linear function given as

(2) $$f\left(x\right)=\left\{\begin{array}{cc} 0, & if\;x\leq 0\\ x, & if\;0<x<1\\ 1, & if\;x\geq 1 \end{array}\right.$$

We tested the MPDL for segmentation of breast tissue into different tissue classes using a twofold cross validation. The balance between the number of unique signatures used to train various tissue types was maintained by sampling uniformly at a random equal number of specific tissue signatures corresponding to each tissue type on the images. Through empirical data analysis, a maximum of 1000 pixels for each tissue signature across all patients was selected. In the twofold cross validation setting, we randomly divided the total patients into two groups. The MPDL was model was trained on each group separately and tested on the other patient group. The efficacy of the MPDL on each patient was evaluated by the performance of the MPDL model trained when that patient belonged to the test group. Through empirical data analysis, a maximum of 1000 pixels for each tissue signature across all patients was selected. To perform a quantitative comparison between the MPDL segmented regions and the radiological ground truth, we defined the radiological ground truth for breast imaging by the EI segmented regions from the DCE images. Clinically, the postcontrast DCE is used for diagnostic evaluation of breast lesions. The DS was used as the overlap evaluation metric.^58^ The DS index was designed to find the similarity between overlapping regions from two objects. Mathematically, DS is defined by the following equation:

(3) $$DS=\frac{2\left(A\cap B\right)}{\left(A\right)+\left(B\right)}$$

where, A and B are the lesion areas obtained by ground truth (EI segmented postcontrast image) and the multiparametric deep learned image, respectively. The EI segmentation was obtained by thresholding the EI contrast image. The threshold was obtained by evaluating the EI contrast MR image histogram, using the mean and a 95% confidence interval. If the images have full overlap, then the DS = 1.0 and if there is no overlap, then the DS = 0.

#### 2.7.3 Comparative methods — multilayer perceptron (MLP) and convolutional neural networks (CNN)

We compared the performance of the SSAE architecture with two other deep learning architectures that do not require full labeling of the input data, multilayer perceptron (MLP), and CNN.^2^, ^59^, ^60^ The deep MLP was implemented with the same architecture as SSAE (five layers with ten nodes each) but trained in a completely supervised fashion to assess the advantage of unsupervised pretraining in SSAE.

Patch‐based two‐dimensional CNN (2D‐CNN) was trained on image patches of size 5 × 5 × N corresponding to each N dimensional tissue signature. The 5 × 5 image patch of a tissue signature corresponds to the immediate 5 × 5 neighborhood of that voxel position. The 2D‐CNN consisted of four layers with 128, 64, 32, and 16 filters, respectively, followed by a fully connected layer and a softmax layer.^61^ Each layer of the 2D‐CNN had the following components:

- Convolutional layer with trainable filters of size 3 × 3
- ReLU activation function given by the following equation

(4) $$f\left(x\right)=\left\{\begin{array}{cc} 0, & if\;x<0\\ x, & if\;x\geq 0 \end{array}\right.$$

- Max pooling layer with a 2 × 2 window.

#### 2.7.4 Multiparametric classification deep learning framework

##### SAE‐SVM architecture

**Figure 3:**
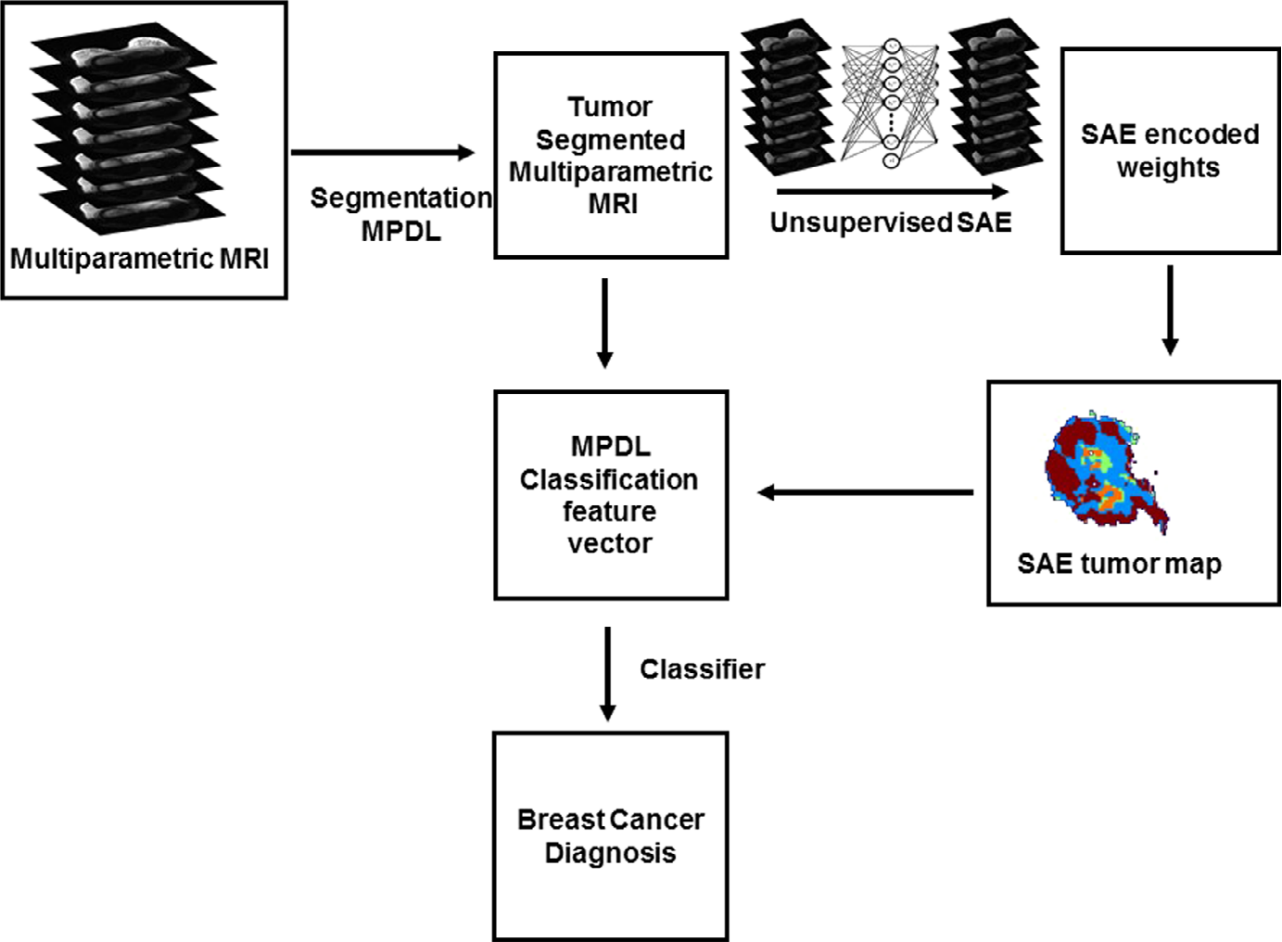
Framework for multiparametric magnetic resonance imaging (mpMRI) classification learning framework trained to classify the segmented tumors from breast mpMRI as benign or malignant. The mpMRI classification deep learning framework is composed of three parts: segmentation from a multiparametric deep learning network to create tumor masks, unsupervised stacked autoencoder to learn a representative feature map for each patient and a hybrid isomap‐support vector machine to produce final classification and diagnosis. [Color figure can be viewed at http://www.wileyonlinelibrary.com]

We developed the mpMRI feature extraction and classification framework termed SAE‐SVM by combining sparse autoencoder (SAE) with support vector machine (SVM) algorithm.^62^ Figure 3 demonstrates the mpMRI classification framework. The first component of the SAE‐SVM is the unsupervised SAE component, which automatically extracts the mpMRI intrinsic tissue signature of each lesion segmented using the segmentation MPDL.

The input to the SAE is represented as $L=\left\{l^{\left(1\right)},l^{\left(2\right)},\ldots ,l^{\left(N\right)}\right\}\in R^{D}$, where D is the dimensionality of the input tissue signatures, N is the total number of voxels in the lesion. The SAE layer transforms input, L, into an encoded intrinsic representation of the lesion, $M=\left\{m^{\left(1\right)},m^{\left(2\right)},\ldots ,m^{\left(N\right)}\right\}\in R^{d}$ where d is the number of nodes in the hidden layer of the SAE. The SAE encoding then transforms the mpMRI of the lesion into an SAE feature map.

The second layer of the SAE component is the statistics layer which characterizes the activation of each node in the SAE feature map with its mean, standard deviation, maximum, and minimum values. The output of statistics layer from each MRI parameter forms a feature vector, *Z*. The feature vectors from each MRI parameter are then concatenated to form an mpMRI classification feature vector, *W*.

The final component of the SAE‐SVM framework is the linear SVM trained on *W* to classify the benign and malignant tumors.

##### Multiparametric MRI classification deep network training and evaluation

For the mpMRI classification, through empirical testing, the training parameters of the SAE were set as: number of nodes in the hidden layer = 10, L2 regularization penalty = 0.001, sparsity proportion = 0.25, sparsity regularization = 4, encoder transfer function: sigmoid, decoder transfer function: linear.

The SAE‐SVM feature extraction and classification method was tested using leave‐one‐out and ten‐fold cross validation with sensitivity, specificity, and area under the receiver operating characteristic curve (AUC) as the evaluation metrics. The imbalance in the number of benign and malignant lesions was resolved by setting a higher misclassification cost for benign than malignant lesions. We determined the optimal value of the misclassification penalty using a grid search on misclassification penalty ratios from the set:

(5) $$\text{Benign}:\text{Malignant}=\left\{1:1,1.5:1,2:1,2.5:1,3:1,3.5:1,4:1\right\}$$

In grid search, each of the different misclassification penalty ratios in the above set are tested in a leave‐one‐out cross validation setting and the misclassification penalty that achieves the maximum AUC was chosen as the optimal misclassification penalty.^63^

#### 2.7.5 Validation of the multiparametric deep learning network

##### Magnetic resonance imaging data

The validation group included 50 patients from UCSF’s I‐Spy ACRIN trial to test our MPDL network.^48^, ^49^, ^50^ To compare with our dataset, we used the baseline DCE contrast imaging sequence from the study. The UCSF data were registered to the precontrast DCE images, and the lesion volumes were calculated. After application of the MPDL to the UCSF data, the MPDL lesion volumes were calculated. The segmented MPDL and UCSF volumes were compared and analyzed for the percent difference between each volume set.

### 2.8 Statistical methods

We calculated the sample size of the training dataset based on the ROC curve.^64^ A sample size of 112 subjects can give 85% power to detect a specificity of at least 80% (under significance level alpha = 5%). The same sample size also gives us greater than 85% power to differentiate sensitivities between 80% and 95% at alpha = 5% significance level. For the validation dataset, a sample size of 44 subjects was estimated using a paired t‐test to give us 90% power with a 5% significance level to test the MPDL model.^65^

We computed summary statistics (mean and standard deviations) from the quantitative imaging parameters from the mpMRI. All lesion area values are presented as mean ± standard deviation. A Student’s two‐tailed *t*‐test was used to determine any statistical significance of the difference between the areas of malignant and benign lesion and the percent difference between boundaries of the malignant and benign lesion. Bland–Altman tests were run to evaluate the agreement between the measurements between the test and validation datasets.^66^, ^67^ Sensitivity and specificity with area under curve (AUC) metrics were computed. Statistical significance was set for *P* < 0.05.

## 3 Results

### 3.1 Quantitative mpMRI

In the training dataset, the DWI and DCE sequences provided quantitative radiological metrics. There were significant differences (*P* < 0.001) between the ADC map values for malignant and benign breast lesions. ADC values for malignant cases were (mean and standard deviation) 1.26 ± 0.13 (mm^2^ × 10^−3^/s) and benign lesions were 1.74 ± 0.17 (mm^2^ × 10^−3^/s). Glandular tissue ADC values for malignant and benign lesions were not significantly different, 2.16 ± 0.46 and 2.34 ± 0.33 (mm^2^ × 10^−3^/s), respectively.

The PK‐DCE values were significantly different (*P* < 0.05) between malignant and benign lesions. The K^trans^ values were 0.55 ± 0.32 (1/min) and EVF was 0.30 ± 0.16 for malignant cases and 0.25 ± 0.19 (1/min) and 0.22 ± 0.13 for benign cases, respectively.

### 3.2 Training dataset

**Figure 4:**
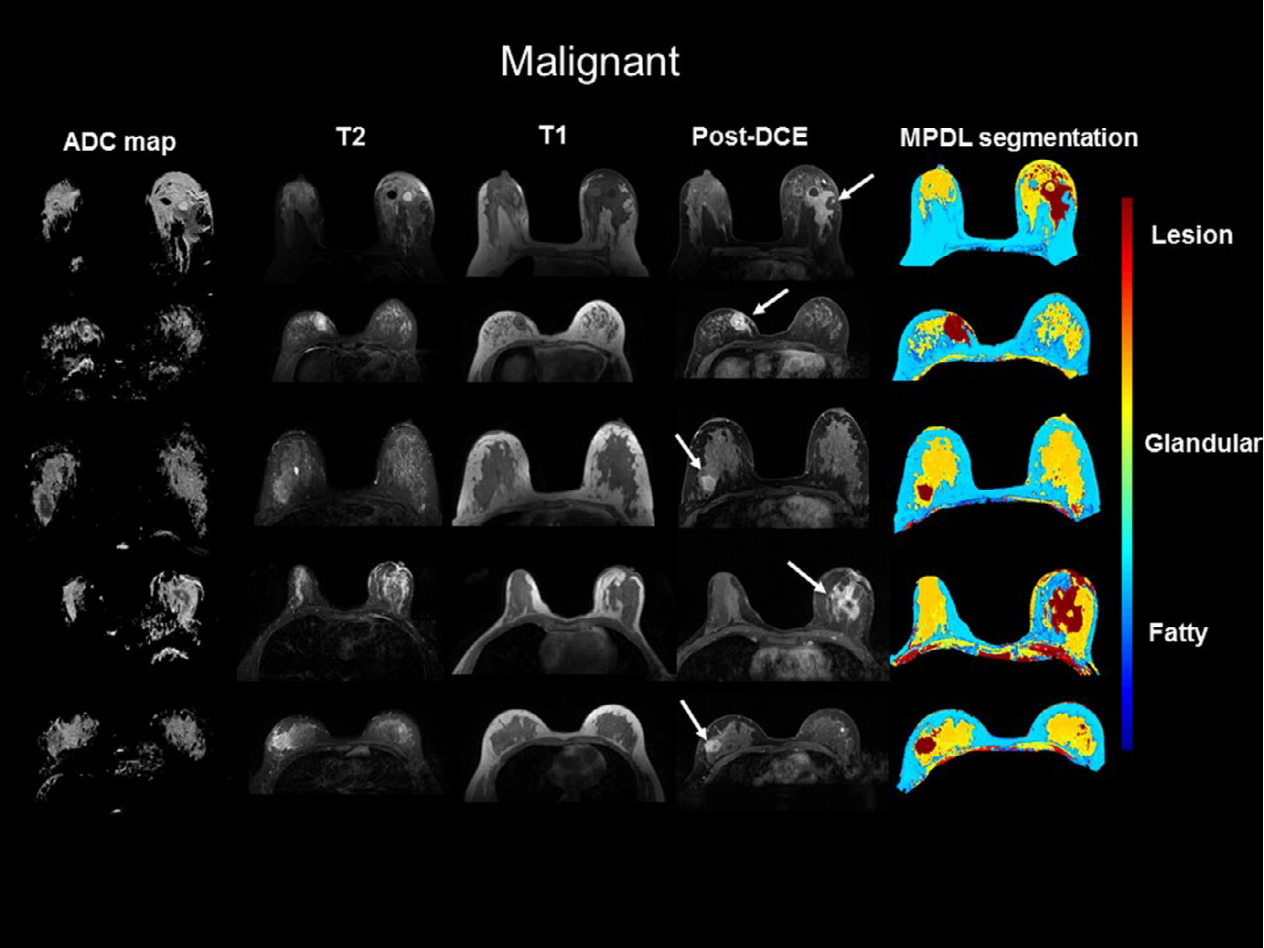
Illustration of the results of multiparametric deep learning (MPDL) network on axial breast magnetic resonance imaging. The MPDL results are shown in five representative malignant lesions. The white arrows show where the lesions are located within the breast. The color coding for different tissue types is shown to the right of the images. [Color figure can be viewed at http://www.wileyonlinelibrary.com]

**Figure 5:**
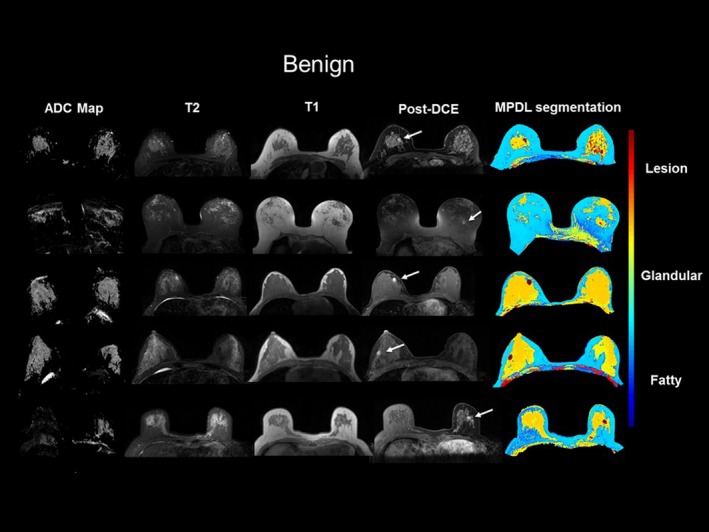
Illustration of the results of multiparametric deep learning (MPDL) network on axial breast magnetic resonance imaging. The MPDL results are shown in five representative benign lesions. The white arrows show where the lesions are located within the breast. The color coding for different tissue types is shown to the right of the images. [Color figure can be viewed at http://www.wileyonlinelibrary.com]

**Figure 6:**
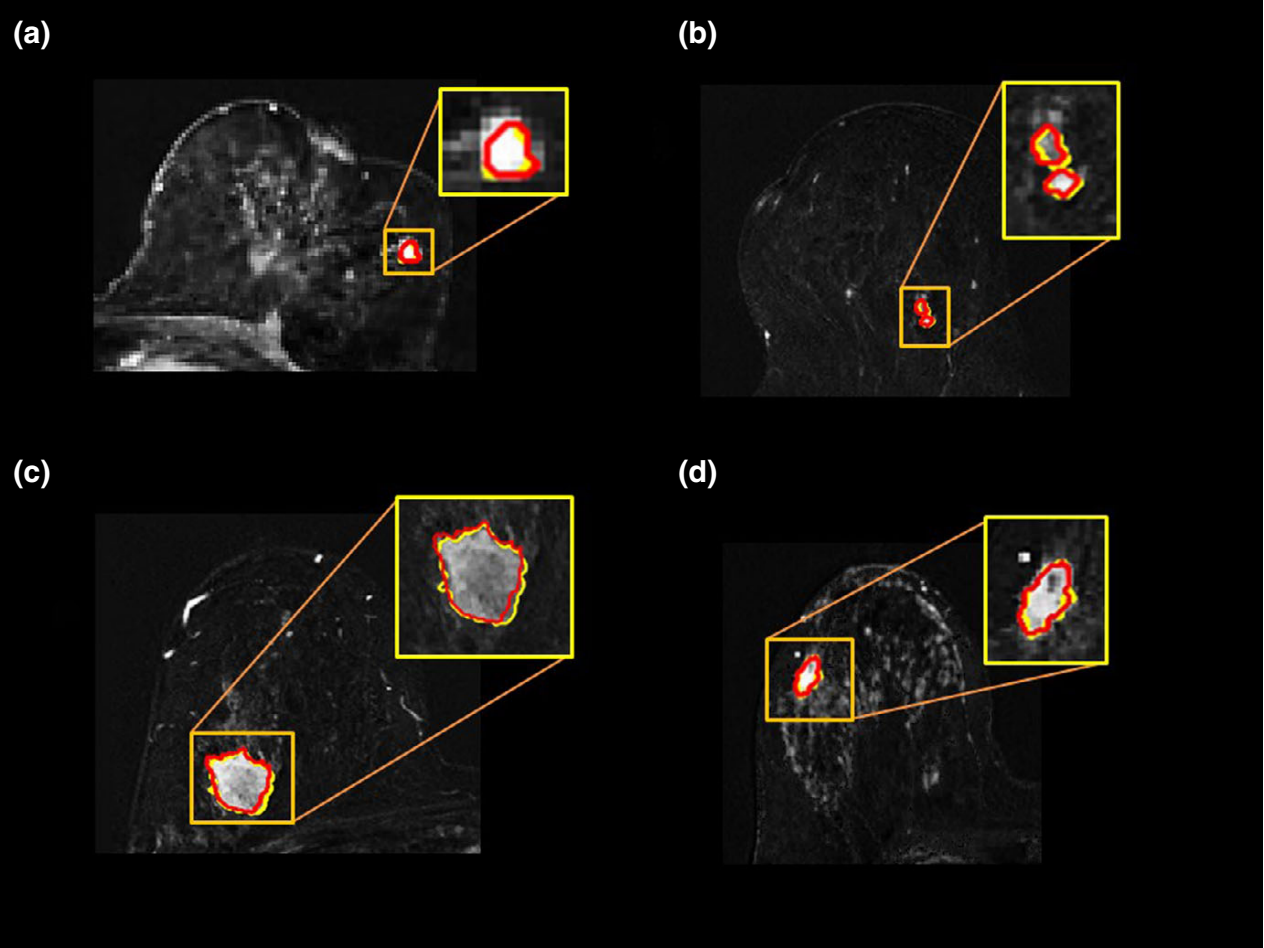
Demonstration of dice Similarity overlap between the Eigenimage and multiparametric deep learning (MPDL) segmentation masks of two benign (A and B) and two malignant (C and D) lesions. The resulting masks are overlaid on the subtracted dynamic contrast‐enhanced image. The Eigenfilter segmentation boundary is shown in yellow, and the MPDL segmentation boundary is displayed in red. [Color figure can be viewed at http://www.wileyonlinelibrary.com]

**Figure 7:**
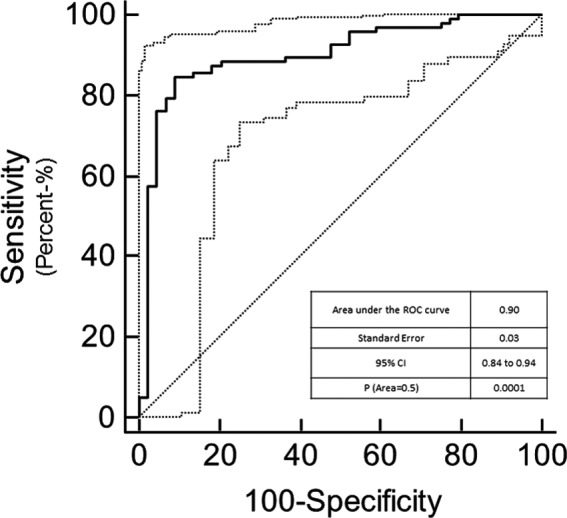
The receiver operating characteristic curve from the SAE‐SVM classification of the multiparametric deep learning segmentation. The SAE‐SVM classification of the lesions demonstrated excellent metrics with a sensitivity of 86% and specificity of 86% and an AUC = 0.90 (95% CI = 0.84 to 0.94 is shown by the dotted lines).

The MPDL tissue signatures were defined for different breast tissue types (Fig. 1) and applied to the 145 mpMRI breast cases. Figure 2 shows the mpMRI deep network for segmentation of the tissue signatures. Figure 4 illustrates the results on five representative patients with malignant lesions. Similarly, Fig. 5 illustrates the mpMRI deep network segmentation results on five patients with benign lesions. Figures 4 and 5, demonstrate the successful segmented breast lesions using the mpMRI deep in patients with either benign or malignant lesions using the tissue signature model. The DS index evaluated in the twofold cross validation setting for the MPDL lesion segmentations demonstrated excellent overlap with a mean and standard deviation (SD) of 0.87 ± 0.05 for malignant and 0.84 ± 0.07 for benign lesions. We show representative cases for each type of lesion in Fig. 6. Finally, the sensitivity and specificity for differentiation of malignant from benign lesions, evaluated in the leave‐one‐out cross validation setting, were 86% and 86%, respectively, with an AUC of 0.90 and shown in Fig. 7. The positive predictive and negative predictive values were 0.93 and 0.73, respectively. The optimal value of misclassification ratio was 2.5:1, where benign lesions had the misclassification penalty set 2.5 times that of malignant lesions.

### 3.3 Validation testing

**Figure 8:**
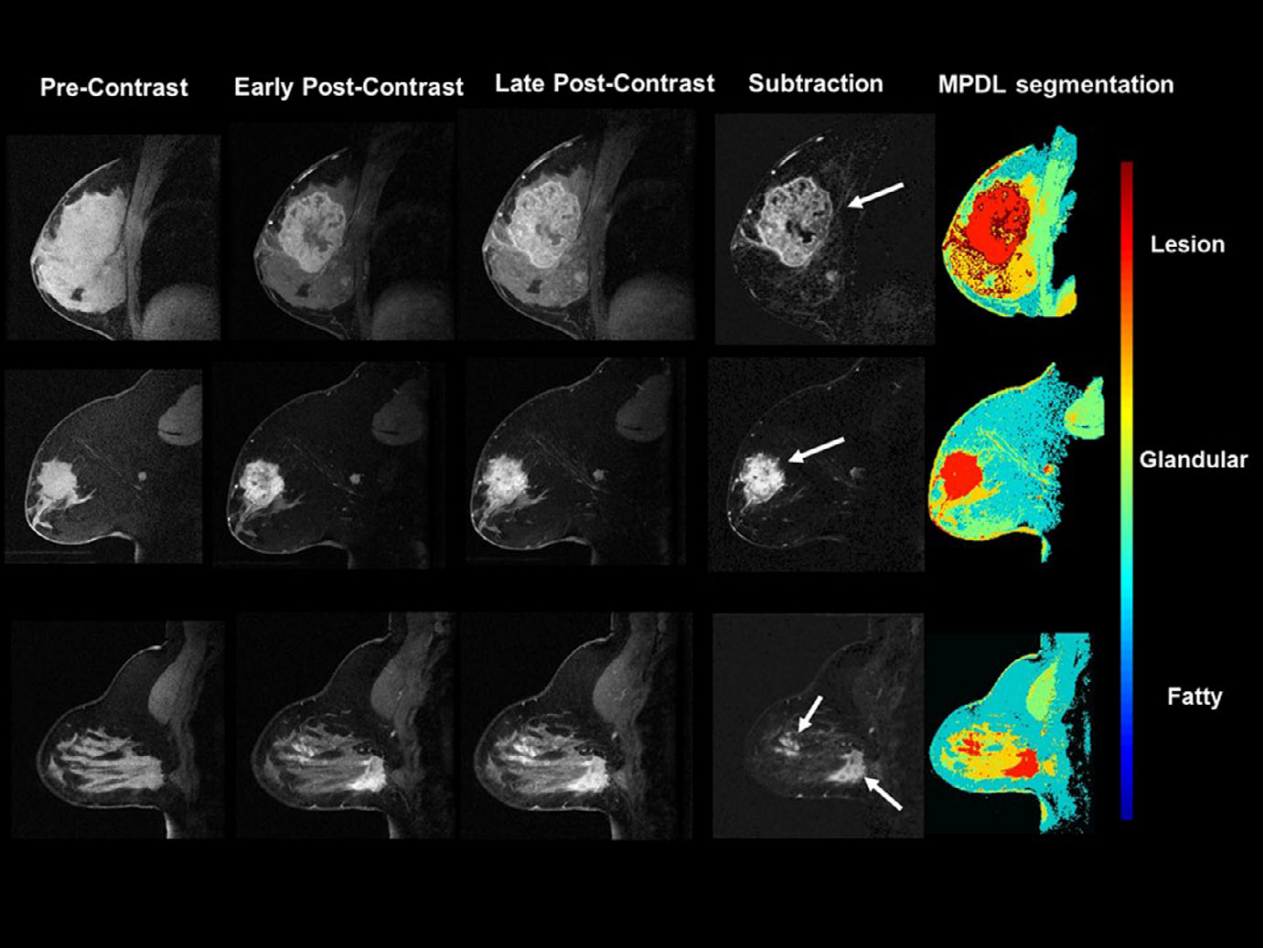
Demonstration of multiparametric deep learning (MPDL) segmentation from the validation cohort using the trained MPDL signatures from the testing dataset. The resulting MPDL segmentations are shown in representative sagittal breast MRI cases. Note, the imaging plane was different in the validation dataset than in the training dataset. The white arrows show the lesion location within the breast. The color coding for different tissue types is shown to the right of the images. [Color figure can be viewed at http://www.wileyonlinelibrary.com]

**Figure 9:**
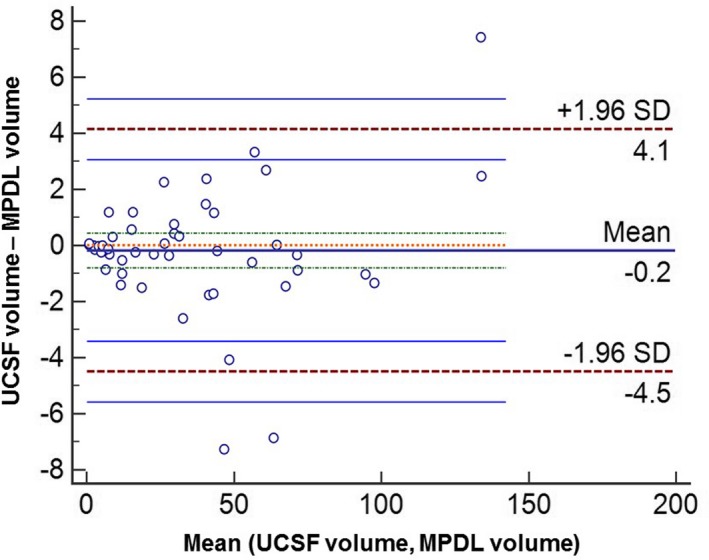
Bland–Altman plots showing the limits of agreement of the two highly correlated (R = 0.99) measurements of the segmented volumes between the validation set from the University of California, San Francisco (USCF) and multiparametric deep learning (MPDL). The mean is shown by the solid horizontal black center line (−0.2) and the red dotted lines show the limits of agreement (LOA). The solid blue lines around the LoA represent the 95% confidence interval of the LoA. [Color figure can be viewed at http://www.wileyonlinelibrary.com]

The validation results from the application of the MPDL tissue signature model to the UCSF independent data set were excellent. The UCSF and MPDL lesion volumes were significantly (*P* < 0.05) correlated with R = 0.998. The percent difference between the datasets was 4.4% ± 3.9%. Figure 8 illustrates segmented lesions from the MPDL and UCSF data showing the accuracy of the MPDL model. The Bland–Altman plot was used to test each volume measurement and shown in Fig. 9, showing excellent agreement between the volume measurements from both the USCF and our dataset.

### 3.4 Comparison to other deep network architectures

**Figure 10:**
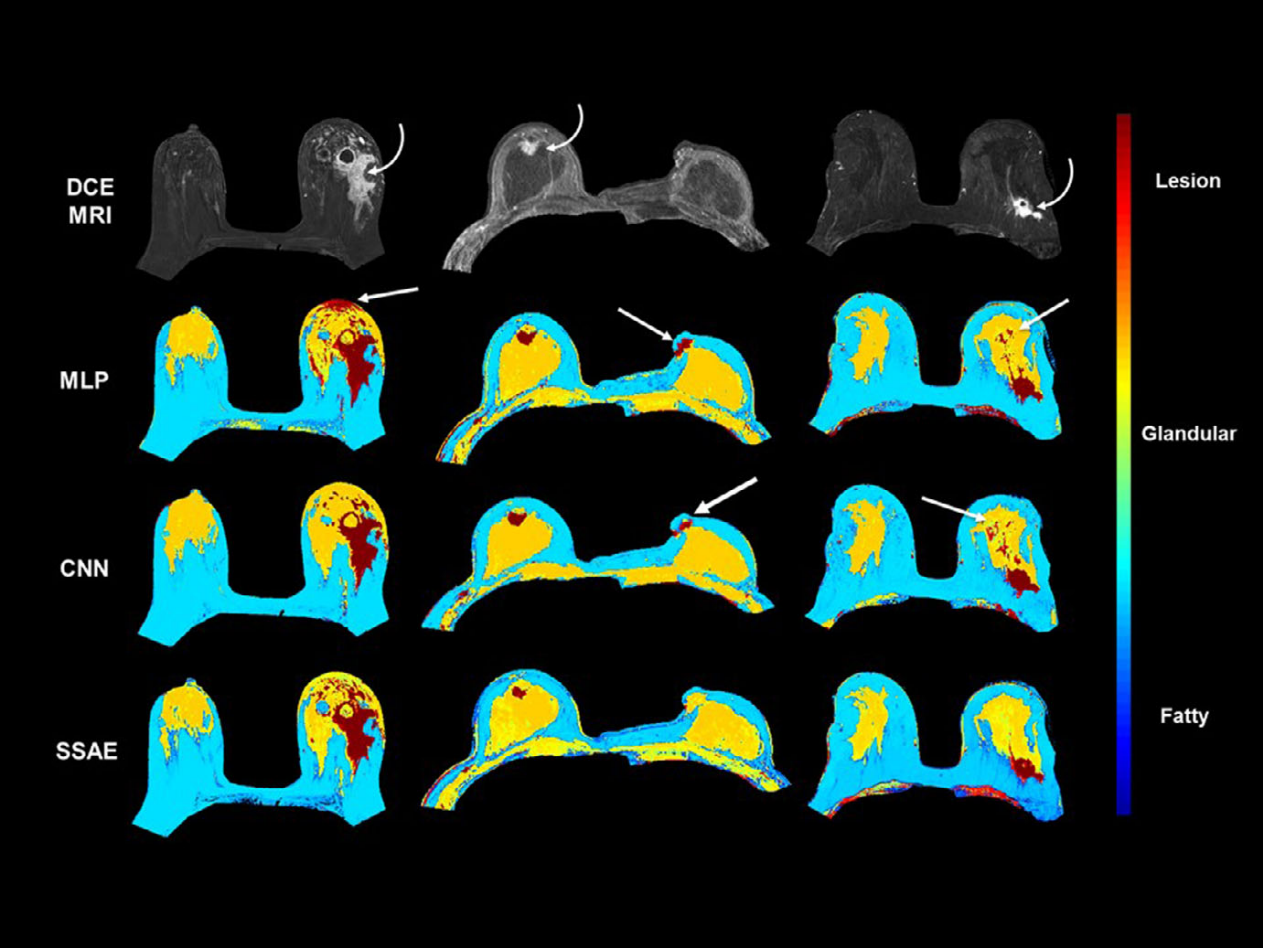
Comparison between different deep learning architectures of multilayer perceptron (MLP), two‐dimensional convolutional neural network (2D‐CNN), and stacked sparse autoencoders (SSAE) in three example patients with the curved white arrows showing the location of the lesions. The segmentations were similar across each of the DL algorithms. However, the time complexity and training were different for each, where the MLP and CNN were costly for both complexity and training. The 2D‐CNN and MLP architectures produced potentially spurious positive regions, as shown by the straight white arrows for the breast segmentations more than SSAE. [Color figure can be viewed at http://www.wileyonlinelibrary.com]

Figure 10 shows the performance of the three deep learning architectures on example patients. As shown in Fig. 10, the segmentations from all the three architectures were very similar, however, the MLP and 2D‐CNN architectures appear to be more prone to potential spurious results. All the deep learning architectures segmented breast tumors with a high DS (MLP: 0.84 ± 0.08, 2D‐CNN: 0.85 ± 0.05, SSAE: 0.89 ± 0.04) on a randomly selected subset of 20 patients (10 benign and 10 malignant lesions) with SSAE outperforming MLP and 2D‐CNN by 5% and 4% respectively.

## 4 Discussion

Using the multiparametric deep learning network, we have developed, validated, and tested a new computing platform that organizes, integrates, and interprets imaging information using an MPDL tissue signature model. The application of the MPDL tissue signature model resulted in excellent segmentation and classification of different breast tissue types. This study used an integrated mpMRI breast deep learning model in a retrospective study and showed that MPDL tissue signatures accurately define benign and malignant lesions. This work demonstrates that deep learning‐assisted unsupervised segmentation using mpMRI signatures can detect heterogeneous zones within breast tissues and lesions. We can use these heterogeneous regions for further classification of breast tissue and lesions by quantitative ADC maps and/or PK‐DCE parameters. Finally, the MPDL model with a machine learning classification distinguished between benign and malignant lesions with excellent sensitivity and specificity.

The results from the application of the MPDL tissue signature model to an independent breast MRI dataset showed the robust nature of the MPDL model. Importantly, the MPDL model could accurately segment breast tissue regardless of the magnetic field strength (3 T for our data and 1.5 T for the validation set). The MPDL model was invariant to the MR imaging orientation, as our dataset was in the axial plane, while they scanned the validation dataset in the sagittal plane. Finally, the MRI parameters and the temporal resolution of the DCE images used to train the MPDL model were different between our dataset and the validation dataset, again reasserting the robust nature of the MPDL model. These data suggest that this may eliminate or reduce the need to retrain the MPDL model in different settings. This invariance is because of the underlying depiction of the tissue using tissue signature vectors, which capture the underlying tissue characteristics and allow for this to change for different MRI inputs. We based the mpMRI parameters used in this study on our and others’ previous results in patients.^24^, ^25^, ^26^, ^68^, ^69^, ^70^ These studies showed that the combined MRI sequences consisting of DWI, ADC, and PK‐DCE were highly correlated with the histological phenotype of the tissue. The sensitivity and specificity of classification between malignant and benign tumors by MPDL were similar to those of radiologists.^22^, ^23^, ^71^ This is very encouraging when future reading rooms will have advanced computing power to assist radiologists in the triage and interpretation of images. We believe, it is very unlikely that machine or deep learning will replace radiologists as has been suggested by some, yet we propose there will be a role for augmenting radiologists’ skills with these tools for improved efficiency and accuracy of interpretation.

Our results demonstrate that we can use the MPDL method on distinct datasets. The I‐Spy ACRIN trial is one of the largest MRI trials and incorporates several MRI field strengths of Ref. [48, 50]. However, the ability of the MPDL to learn different tissue signatures allows it to adapt to diverse datasets with highly accurate results. We demonstrated this with the high segmentation accuracy of the validation dataset using different input MRI data. The MPDL algorithm outperformed the multilayer perceptron (MLP) with exactly the same architecture demonstrating the effectiveness of unsupervised pretraining in datasets with sparse input. Furthermore, the MPDL algorithm also compared favorably to the patch‐based 2D‐CNN algorithm. The number of trainable parameters in a 2D‐CNN architecture was significantly higher than the number of trainable parameters for MPDL. As a result, the size of the training dataset was not sufficient to optimally train the CNN, producing suboptimal results with the CNN resulting in potentially spurious segmentations. Using the MPDL allows the use of sparse datasets with tissue signature vectors to accurately define different tissue types without the need of a large training set, which is encountered in most clinical settings.

Our results are consistent with previous reports segmenting the breast lesion using deep learning methods and breast MRI.^33^, ^36^, ^37^, ^38^, ^39^, ^40^ However, most of those studies used at most one MRI sequence, specifically, the DCE and some used two sequences, either adding a T_1_‐weighted or DWI sequence. All the previous reports utilized a fully annotated dataset, whereas our dataset was defined by the intrinsic tissue signature from all the sequences, similar to how a radiologist views MRI, and our method outperformed the quantitative metrics presented in the previous reports. Moreover, we used an independent dataset to confirm the MPDL method and demonstrated the potential generalizability to other breast cancer centers.

There are, however, technical limitations to the use the MPDL network in practice. First, increased computational power on the graphical processor units [(GPU) > 2500 cores, >12 GB)] used here may not be widely available. But, the use of advanced DLN with GPU computing is rapidly finding applications in many radiological datasets.^14^, ^15^, ^17^, ^18^, ^19^, ^72^ More specific to the present study, any assessment of the clinical value of a MPDL network will require additional studies in a larger patient population with a prospective trial with subsequent follow‐up and pathological correlation. This would provide us with new data to explore the exact application of the MPDL model to larger studies.

In conclusion, we have demonstrated that an integrated MPDL method accurately segmented and classified different breast tissues types from multiparametric breast MRI. The MPDL images allow for improved visualization of different tissue characteristics based on multiple radiological parameters**.**

### Code Availability

Our software will be freely available to academic users after issue of pending patents and a materials research agreement is obtained from the university. Due to University regulations, any patent pending software is not available until a patent is issued.

### Data Availability

All relevant clinical data are available upon request with adherence to HIPPA laws and the institutions’ IRB policies.

## Authors' Contributions

MAJ and VP developed the concept, algorithm, and performed the testing, statistical methods, and manuscript writing and review. DB, RE, SH, IK, and KM performed the data acquisition, analysis, and manuscript writing and review.

## Conflict Of Interest

The authors declare no competing interests or no conflicts.

## Figures and Tables

**Figure A1:**
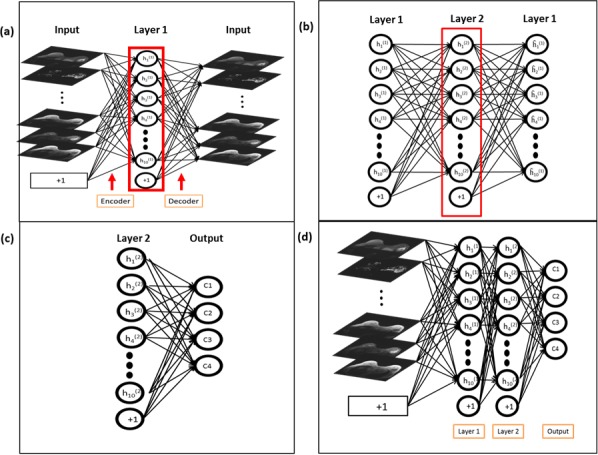
(a) An example autoencoder architecture of learning intrinsic representation of the input mpMRI. (b) The autoencoders used to pre‐train each subsequent layer in the stacked sparse autoencoder (SSAE) architecture (c) The softmax classifier is used to classify the final layer into four classes–C1, C2, C3, and C4. (d) The complete two‐layer SSAE architecture is shown for this example. [Color figure can be viewed at http://www.wileyonlinelibrary.com]

## Appendix 1

The MPDL framework is based stacked autoencoders and shown in Fig. 2. An autoencoder has two parts: an encoder and a decoder. The encoder maps the vector X to the vector h^(1)^ representing the hidden layer as follows:

(A1) $$h^{\left(1\right)}=f\left(\mathrm{WX}+b\right)$$

where *f* is the transfer function for the encoder, is the matrix of weights, and *b* is the bias vector. Where the decoder maps h^(1)^ back to X using the following equation:

(A2) $$\hat{X}=g\left(W^{{}^{\prime }}h^{\left(1\right)}+b^{{}^{\prime }}\right)$$

The values of *W’* and *b’* are equal to the transpose of *W* and *b* in case the weights are tied between the encoder and decoder. Figure A.1(a) demonstrates an autoencoder for reconstructing mpMRI input tissue signatures. The encoder–decoder architecture is used to pretrain the hidden layer. Once the hidden layer weights are optimized, the next hidden layer is pretrained by attempting to reconstruct the output of the previous encoder (hidden layer) as shown in Fig. A.1(b). The complete stacked sparse autoencoder was constructed by pretraining and stacking each encoder in the network followed by a softmax layer for classification as shown in Figs. A.1(c) and A.1(d).

The nodes within the hidden layer were further specialized for activation in response to a subset of the total number of MPDL tissue signatures using sparsity regularization. For example, after training the sparse autoencoder on an mpMRI dataset, node one may have “specialized” in activating only in response to a fatty tissue signature. While node two may be “specialized” in activating only in response to the glandular tissue signature. Mathematically, the average activation, $\hat{\rho_{j}}$ of a neuron, j is given by the following equation

(A3) $$\hat{\rho_{J}}=\frac{1}{N}\sum_{i=1}^{N}h_{j}^{\left(1\right)}\left(x_{i}\right)$$

where N is the total number of training samples. If *ρ* denotes the desired average activation or the sparsity proportion of the neuron, j across all the training samples, the goal is to impose the constraint $\hat{\rho_{J}}=\rho$. The sparsity regularization term added to the cost function is given as

(A4) $$R_{S}=\beta \sum_{j=1}^{d}\left(\rho \log\hat{\rho_{j}}+\left(1-\rho \right)\log\frac{1-\rho }{1-\hat{\rho_{j}}}\right)$$

where *β* is the sparsity regularization penalty. Because we have four tissue classes, the sparsity proportion, *ρ* was set at 0.25 and the penalty, β was set at 4 to train each sparse autoencoder.^44^, ^45^, ^57^

The output of the final sparse autoencoder is used as input for training a softmax classifier to classify the tissue signatures as background, fat, glandular, or lesion. The cost function for training the softmax layer of the mpMRI segmentation deep network is based on cross entropy, given by

(A5) $$J=\frac{1}{N}\sum_{i=1}^{N}\sum_{j=1}^{4}t_{\mathrm{ij}}\;\ln\;y_{\mathrm{ij}}+\left(1-t_{\mathrm{ij}}\right)\ln\left(1-y_{\mathrm{ij}}\right)$$

where *t_ij_* is the target class and *y_ij_* is the output of the deep network at the softmax classification layer. Figure A.1 demonstrates an example two‐layer stacked sparse autoencoder.

The weights of the pretrained network were further fine‐tuned using a scaled conjugate gradient backpropagation method to improve the classification accuracy of the pretrained network. The pretrained weights are especially useful when the application is limited by the number of training examples. After training, the first layer of the SSAE learns the most representative tissue signatures from the input dataset, while the stack of subsequent autoencoders forms a composite representation of the tissue signatures, such that each node of the final layer specializes in recognizing tissue signatures from a single tissue type.
